# Intra-peritoneal mature benign cystic teratoma deposits: an unusual complication from previous surgical resection of an ovarian mature benign cystic teratoma

**DOI:** 10.1259/bjrcr.20180008

**Published:** 2018-07-10

**Authors:** Radhika Prasad, Cyril Sieberhagen, Stephen Fenwick, Ashok Katti, Rob Davis

**Affiliations:** 1 Department of Radiology, Aintree University Hospital NHS Foundation Trust, Liverpool, UK; 2 Department of Gastroenterology, Aintree University Hospital NHS Foundation Trust, Liverpool, UK; 3 Department of Surgery, Aintree University Hospital NHS Foundation Trust, Liverpool, UK

## Abstract

In this case, we describe multiple unusual intra-peritoneal lesions containing fat, soft tissue and calcification. These were found radiologically and histologically to be in keeping with mature benign cystic teratoma intra-peritoneal deposits, also described as dermoid cysts. These were presumed to be due to seeding from surgical resection of ovarian mature benign cystic teratoma 5 years previously. This surgical complication has not been well documented thus far in the literature.

## Clinical presentation

A female patient in her early forties presented to the gastroenterology team with recurrent upper abdominal pain.

## Investigations/Imaging findings

**Figure 1.**
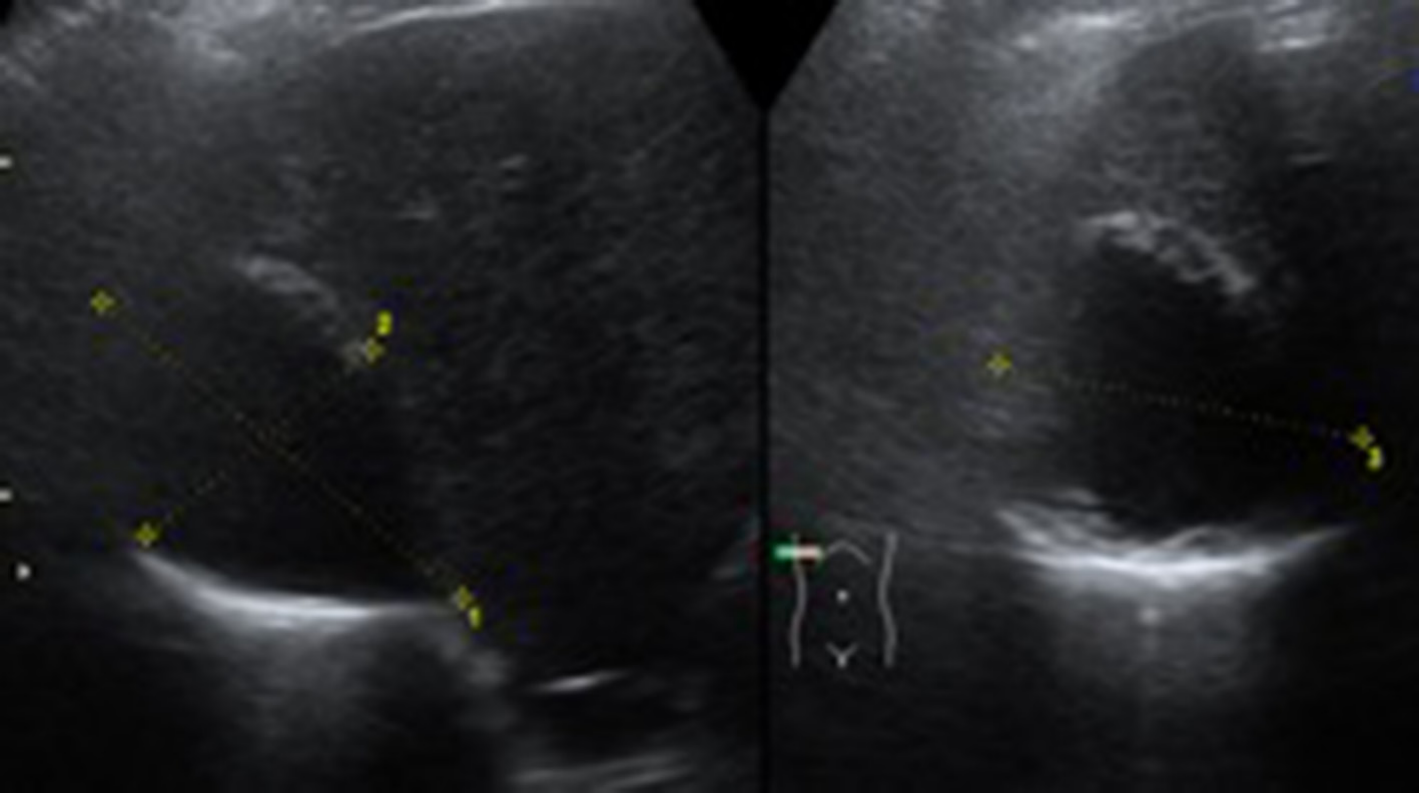
Ultrasound shows right upper quadrant masses with hypoechoic and hyperechoic components.

An initial abdominal ultrasound found three hypoechoic septated masses in the right upper quadrant. The largest, measuring 70 × 44 × 56 mm, had a thickened hyperechoic wall. It was unclear whether the masses originated from the liver or not at this point. (Figure 1).

**Figure 2.**
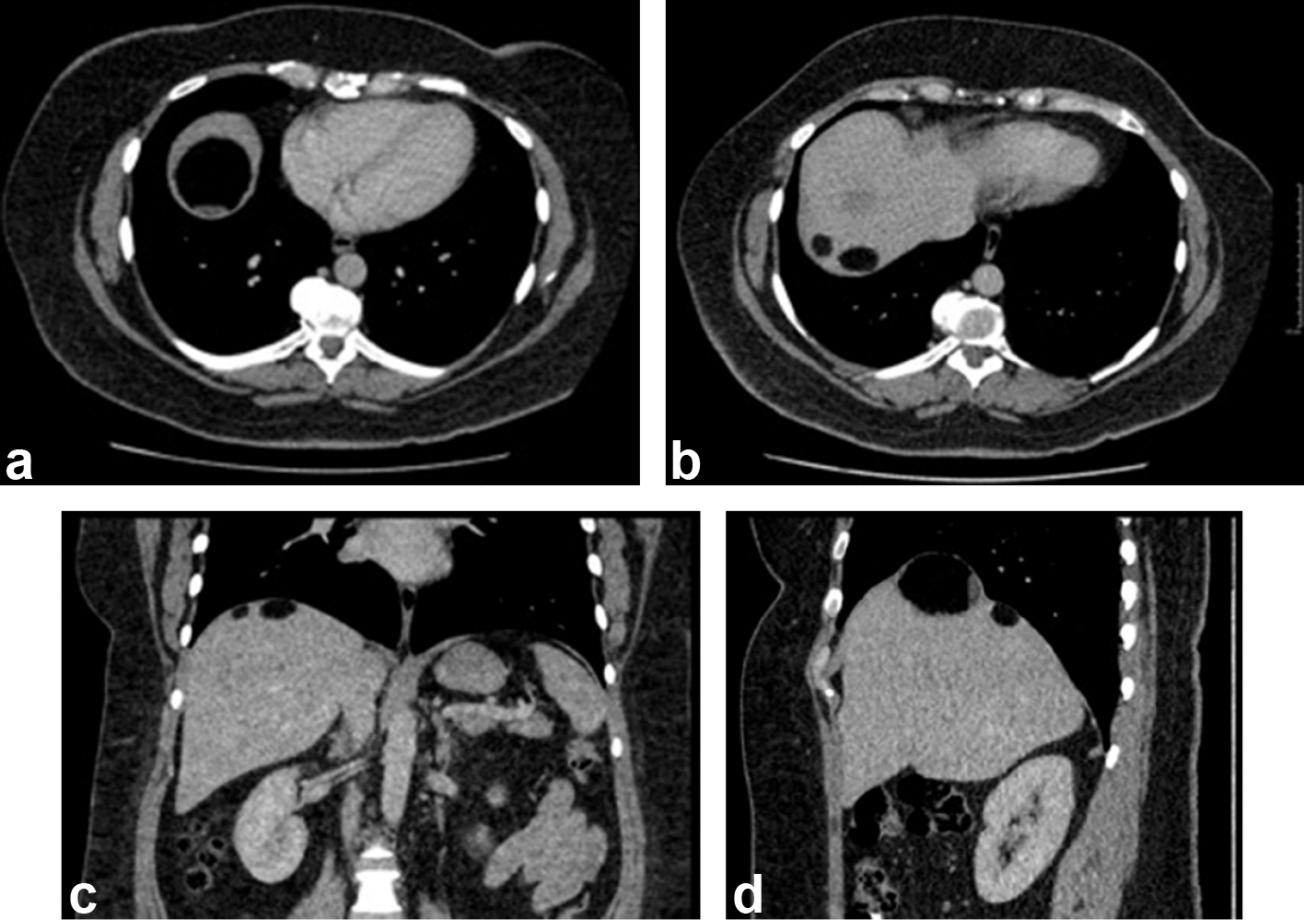
(a, b) Axial, (c) coronal and (d) sagittal: these images from CT abdomen in portal venous phase shows right upper quadrant lesions containing macroscopic fat, soft tissue and calcification.

**Figure 3.**
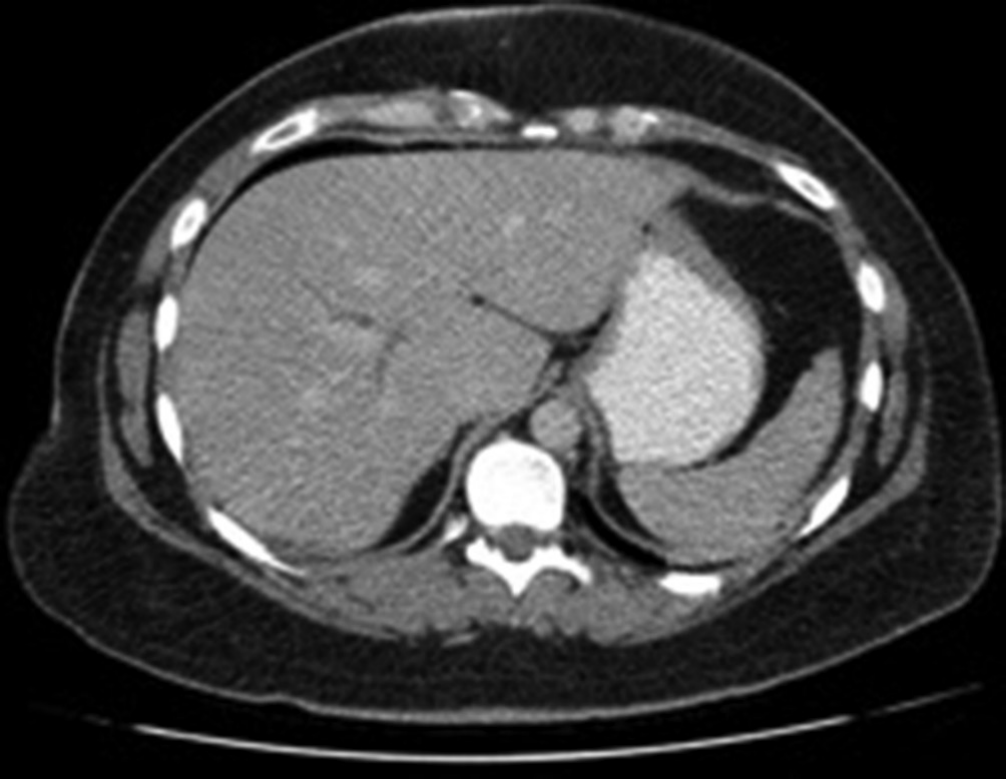
CT abdomen 5 years prior show no liver lesions.

**Figure 4.**
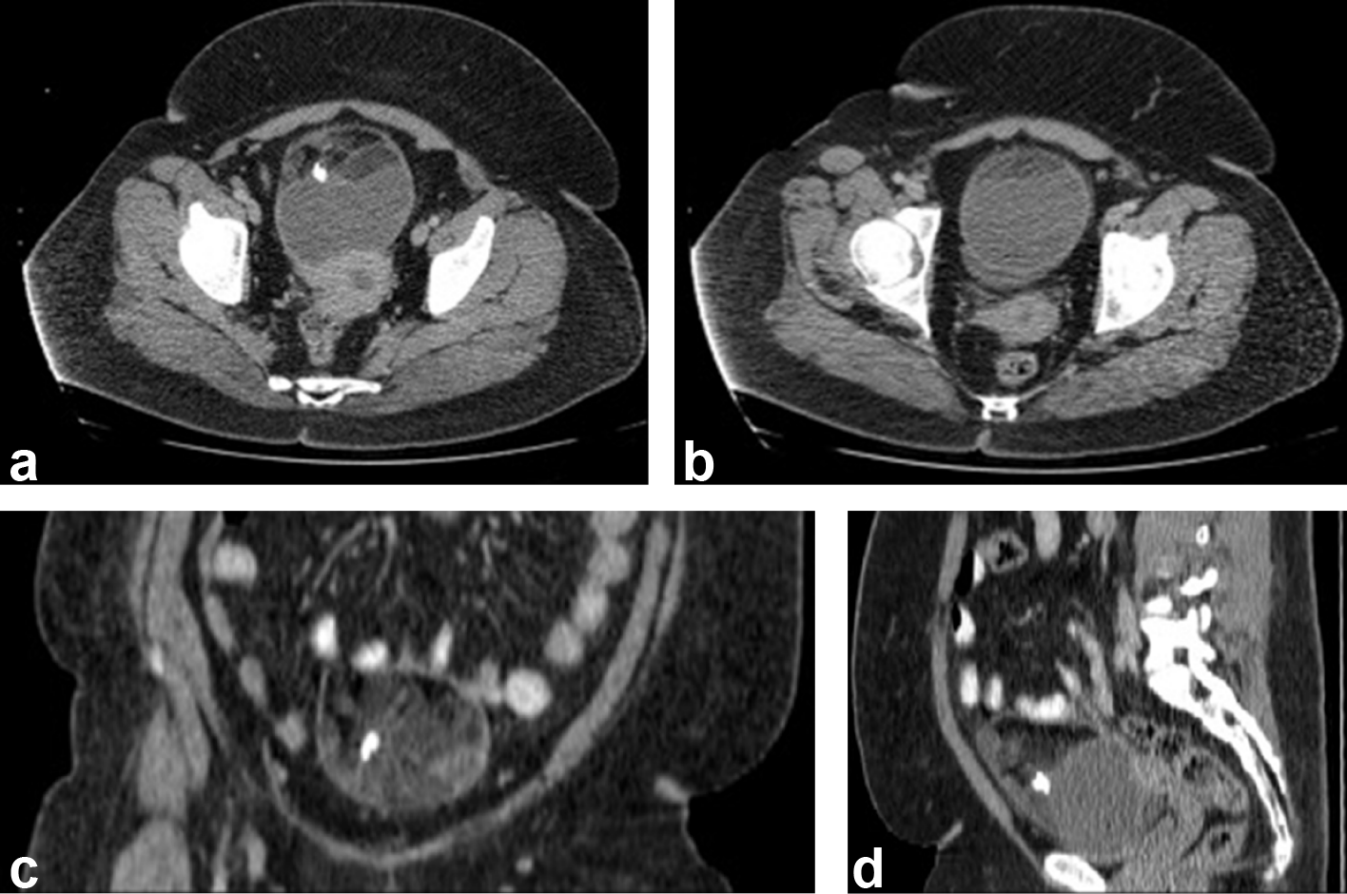
(a, b) Axial, (c) coronal and (d) sagittal. These CT abdomen image 5 years prior shows a 10 cm ovarian mature benign cystic teratoma.

An abdominal contrast-enhanced CT was organised to further characterise these lesions. This demonstrated multiple mixed-density lesions along the liver surface containing fat, soft tissue and calcification (Figure 2a–d). When previous imaging was reviewed, a CT abdomen and pelvis performed 5 years prior to this presentation demonstrated a normal liver with no focal lesion (Figure 3), as well as a 10 cm ovarian mature cystic teratoma, containing macroscopic fat, soft tissue and calcification (Figure 4a–d). Shortly after the patient underwent laparotomy to resect this.

**Figure 5. a and b.**
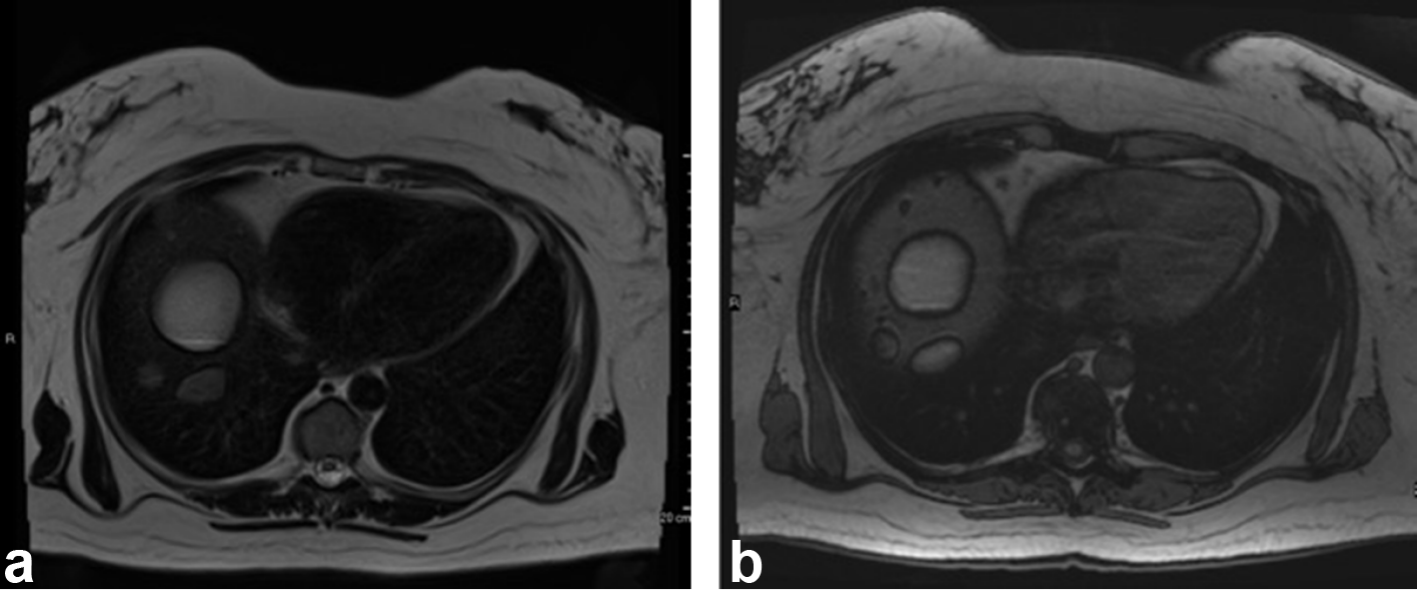
(a, b) MR liver. (a) A *T*_2_ showing high signal in the right upper quadrant lesions. (b) A *T*_1_ out of phase image, showing high signal within the right upper quadrant lesions with chemical shift artefact *i.e.* macroscopic fat.

A subsequent MR liver showed the lesions were deposited along the liver surface in the right subdiaphragmatic region and adjacent to the inferior vena cava. They displayed high *T*_1_ and *T*_2_ signal, demonstrated chemical shift artefact and suppression on short tau inversion-recovery (STIR)  sequences, confirming that the lesions contained macroscopic fat. There was no abnormal enhancement. (Figure 5a,b).

The case was discussed in hepatobiliary multidisciplinary team meeting at our tertiary teaching hospital.

## Differential Diagnosis

The ultrasound, CT and MR confirmed several intra-peritoneal lesions containing macroscopic fat, soft tissue and calcification.

Initially, it was difficult to ascertain whether these lesions were within or outside of the liver. In the liver, both benign and malignant lesions can contain macroscopic fat. Benign fat-containing liver lesions are lipoma, adenoma and angiomyolipoma. Malignant fat-containing liver lesions are hepatocellular carcinoma, metastases, and primary and secondary liposarcoma.^1, 2^

In the peritoneum, common fat-containing lesions include benign lesions such as lipoma, lipoblastoma, myelipoma, angiomyolipoma. Malignant fat-containing intra-abdominal lesions are liposarcoma and malignant teratoma.^3^

The lesions in this particular case were intra-peritoneal rather than intrahepatic, and as mentioned contained soft tissue and calcification as well as macroscopic fat. The imaging features are typical for mature benign cystic teratoma, although the intra-peritoneal location is rare.

Mature benign cystic teratomas contain tissues with endodermal, mesodermal and ectodermal origins. They are the most common benign ovarian tumour in patients under 45.^4^ Classically, they contain sebum-filled cyst cavity and a protuberance into the cavity containing teeth, hair, adipose tissue or bone is known as a Rokitansky nodule. On imaging, these lesions can vary from a purely cystic lesion, to a mixed-density mass with all three embryonic germ cell layers.^5^

Mature benign cystic teratomas are usually found in the ovary, where common complications are rupture, torsion or malignant transformation. Teratomas can occur in other locations; intra-cranially, intra-orbitally, in the mediastinum, in the sacrococcygeal region, and in the testes.^6^

The imaging features, along with the patient’s relevant surgical history, led to a radiological diagnosis of intra-peritoneal mature benign cystic teratomas from seeding during resection of an ovarian mature benign cystic teratoma.

## Outcome

Due to abdominal discomfort caused by the lesions, the patient opted for the lesions to be surgically resected.

At laparotomy, the findings were of a large cyst involving the right hemidiaphragm, adherent to the adjacent segment 8 of the liver, with smaller surrounding cysts. There were further small cystic deposits adjacent to the right adrenal gland, overlying the right perinephric tissue and on the porta hepatis. A partial resection of the right hemidiaphragm along with the adjacent liver was performed, and all other areas of cystic disease were excised. The diaphragm was repaired using a porcine collagen mesh. The patient made a good recovery from surgery and was discharged home on day 6.

Histology of the lesions confirmed the presence of several mature benign cystic teratomas with densely laminated fibrotic walls, the luminal surface of which displayed a fat necrosis reaction, and occasional coarse calcifications. There was no evidence of neoplasia in the samples.

The lesions were, in conclusion, thought to be due an exaggerated reaction to a previously ruptured mature benign cystic teratoma, possibly intra-operatively. No specific complications were noted in the surgical note from the initial ovarian mature benign cystic teratoma cyst resection laparotomy in 2010.

## Discussion

A literature search of cases of mature benign cystic teratoma deposits was undertaken. (Literature search carried out in December 2015 and updated in December 2017) Medline, Embase and CInahl were searched for the following search: Dermoid cyst/OR dermoid OR “mature cystic teratoma” AND (“drop deposit” OR “secondary site” OR metastases OR spread) AND (peritoneal OR intra-abdominal OR mesenteric).

The search obtained 60 results. A conference abstract by Flack et al^7^ describes a case in which a female patient presenting with abdominal pain was found to have a concurrent ovarian mature benign cystic teratoma and subcapsular fat containing lesions found to be consistent with mature cystic teratomas histologically. However, in our case the initial CT performed before the ovarian mature benign cystic teratoma resection did not show concurrent peritoneal lesions and so the presumption is that this seeding occurred in surgery. Another case by Sho et al^8^ describe a single port site metastasis from previous ovarian mature benign cystic teratoma resection. Our case describes multiple intra-peritoneal deposits.

A few cases describe peritoneal strumosis and struma ovarii.^9^ Struma ovarii is a highly specialised form of mature ovarian teratoma consisting of thyroid tissue. Peritoneal strumosis is when there is spread of mature thyroid tissues into the peritoneal cavity. The lesions we have described histology did not contain thyroid tissue.

In this case, we have described an unusual case of mature benign cystic teratoma deposits thought to be related to seeding from a previous mature benign cystic teratoma resection. A multidisciplinary team approach with the radiology, pathology, gastroenterology and hepatobiliary surgery teams led to the diagnosis.

Mature benign cystic teratoma deposits in the peritoneum should be considered in patients with intra-peritoneal lesions, who have a history of surgical resection of an ovarian mature benign cystic teratoma.

## Learning points

1. Understand the ultrasound, CT and MR features of mature benign cystic teratoma.
2. Understand that intra-peritoneal mature benign cystic teratoma can be an unusual complication of ovarian mature benign cystic teratoma resection.
3. Understand the importance of previous imaging as well as a patient’s previous medical and surgical history.
